# Role of Oxidative Stress in Reperfusion following Myocardial Ischemia and Its Treatments

**DOI:** 10.1155/2021/6614009

**Published:** 2021-05-18

**Authors:** Mi Xiang, Yingdong Lu, Laiyun Xin, Jialiang Gao, Chang Shang, Zhilin Jiang, Hongchen Lin, Xuqin Fang, Yi Qu, Yuling Wang, Zihuan Shen, Mingjing Zhao, Xiangning Cui

**Affiliations:** ^1^Department of Cardiovascular, Guang'anmen Hospital, China Academy of Chinese Medical Sciences, Beijing, China; ^2^First Clinical Medical School, Shandong University of Chinese Medicine, Shandong, China; ^3^Key Laboratory of Chinese Internal Medicine of Ministry of Education, Dongzhimen Hospital, Beijing University of Chinese Medicine, Beijing, China

## Abstract

Myocardial ischemia is a disease with high morbidity and mortality, for which reperfusion is currently the standard intervention. However, the reperfusion may lead to further myocardial damage, known as myocardial ischemia/reperfusion injury (MI/RI). Oxidative stress is one of the most important pathological mechanisms in reperfusion injury, which causes apoptosis, autophagy, inflammation, and some other damage in cardiomyocytes through multiple pathways, thus causing irreversible cardiomyocyte damage and cardiac dysfunction. This article reviews the pathological mechanisms of oxidative stress involved in reperfusion injury and the interventions for different pathways and targets, so as to form systematic treatments for oxidative stress-induced myocardial reperfusion injury and make up for the lack of monotherapy.

## 1. Introduction

Myocardial ischemia is the most frequent form of cardiovascular disease with high morbidity and mortality [1], for which timely restoration of blood flow to the ischemic myocardium (reperfusion) is indispensable for a better patient outcome [2]. However, this reperfusion may cause further myocardial ischemia/reperfusion injury (MI/RI) which leads to cardiac dysfunction such as myocardial stunning, reperfusion arrhythmia, myocyte death, and endothelial and microvascular dysfunction including the no-reflow phenomenon, inflammatory response [3, 4], and other myocardial tissue injury more terrible than that caused by the original ischemic insult [5]. Lethal reperfusion injury, according to a report, accounts for up to 50% of the final myocardial infarct size [5]. Among the complex system networks involved in the pathological mechanisms of MI/RI, such as oxidative stress [6], inflammatory response [7], calcium overload [8], and mitochondrial dysfunction [9], one of the most important pathological mechanisms is oxidative stress (OS) [10].

OS refers to an imbalance between normal oxidant scavenging enzyme systems, such as superoxide dismutase, catalase, and glutathione, and intracellular reactive oxygen species (ROS) production, which leads to toxic accumulations of reactive oxygen intermediates like hydrogen peroxide (H_2_O_2_) [11, 12]. Under physiological condition, ROS are produced as a result of normal cellular metabolism processes, maintaining a dynamic balance with antioxidants [12]. But ROS can also be generated in both ischemia [13–15] and reperfusion period [16, 17]. Limited oxygen availability during the ischemic period is associated with acidosis, energy depletion, and alterations of ion homeostasis, leading to cardiac dysfunction and ultimately cell death [18]. In the presence of residual oxygen, ROS are produced in the myocardium [18], which are attributed to the decrease of endogenous ROS scavenger and increase of ROS production by several mechanisms [13, 19–21]. Much higher levels of oxygen free radical (OFR) production are induced immediately following reperfusion due to the sudden reintroduction of high oxygen tensions [19], leading to oxidative damage of cell structures, such as initiating lipid peroxidation, protein carbonylation, and DNA oxidation [22]. However, many traditional antioxidants do not show significant efficacy [23, 24]. The formation and development of oxidative stress after reperfusion and how it affects injuries involve multiple mechanisms, so developing interventions that act on specific pathways rather than simple antioxidants may be a promising therapeutic approach.

Here, through summarizing pathogenesis of oxidative stress involved in MI/RI, including sources of ROS, ROS-mediated MI/RI, and its related pathways and signaling molecules, as well as various interventions targeting them, we expect systematic treatments against oxidative stress-induced myocardial reperfusion injury to be formed, complications like arrhythmias, myocardial stunning, microvascular obstruction and myocardial remodeling to be inhibited, and mortality following reperfusion to be reduced.

## 2. Sources of ROS

**Figure 1:**
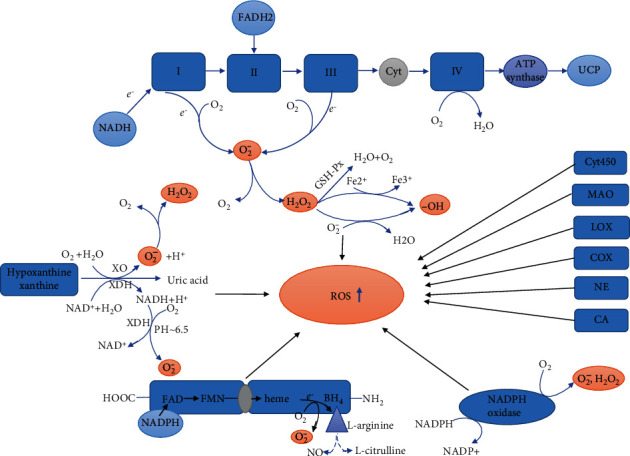
Multiple sources of reactive oxygen species during reperfusion following myocardial ischemia. These are mainly mitochondria, xanthine oxidoreductase, uncoupled nitric oxide synthase, nicotinamide adenine dinucleotide phosphate oxidase, and some other sources. Abbreviations: NADPH: nicotinamide adenine dinucleotide phosphate; NADH: nicotinamide adenine dinucleotide; FADH2: flavin adenine dinucleotide hydrogen transmitter; O_2_^−^: superoxide; Cyt: cytochrome; UCP: uncoupled protein; GSH-Px: glutathione peroxidase; XDH: xanthine dehydrogenase; FAD: flavin adenine dinucleotide; FMN: flavin mononucleotide; BH4: tetrahydrobiopterin; MAO: monoamine oxidase; LOX: lipoxygenases; CA: catecholamine; NE: neutrophil; COX: cyclooxygenase; XO: xanthine oxidase.

Amounts of potential sources of ROS in the postischemic heart are most attributed to one or more enzymes like xanthine oxidase [25], NADPH oxidase (NOX) [26, 27], mitochondria [28], and uncoupled nitric oxide synthase [29], which have been deemed as the most likely causes to oxidative stress during reperfusion and thus the most promising targets for therapeutic measures against reperfusion-induced organ dysfunction and tissue damage [30]. In addition to the above mentioned data, there are also some descriptions about other sources such as monoamine oxidases, lipoxygenases, cyclooxygenases, the cytochrome P450, neutrophils, and catecholamine [4, 31–34]. We summarized the various sources of ROS (Figure 1).

### 2.1. Mitochondria

Mitochondria are deemed the major intracardiac source of ROS RI, impaired autopha/RI [35]. There are at least eleven different sites that associate with substrate catabolism and the electron transport chain (ETC) in mammalian mitochondria generating superoxide and/or hydrogen peroxide [18]. Mitochondrial ROS production involves oxidative phosphorylation linked to aerobic respiration within the mitochondrial ETC [36]. This mechanism has been detailedly described by Cadenas [18]. Electrons are released to cofactors such as NADH and FADH2 via oxidation of substrates and then flow sequentially through a series of redox carriers in respiratory chain complexes, finally reducing oxygen to water with the catalysis by cytochrome c oxidase (CcO). However, at seven different sites along the respiratory chain, electrons derived from NADH or some other donor can directly react with oxygen and generate O_2_^−^ [37], that is electron leakage from the ETC at complexes upstream of CcO, primarily at complexes I and III, causing partial reduction of molecular oxygen to O_2_^−^ instead of reduction to H_2_O [18], and, among these, reverse electron transport at complex I is the main source of superoxide upon reperfusion of ischemic tissue [38, 39]. O_2_^−^ can be converted into H_2_O_2_ and O_2_ either spontaneously or enzymatically catalyzed by superoxide dismutase (SOD) [18, 40]. Hydrogen peroxide (H_2_O_2_) can be fully reduced to water or partially reduced to the hydroxyl radical (•OH) [18]. H_2_O_2_ oxidizes Fe^2+^to Fe^3+^ to generate hydroxyl radicals through the Fenton reaction [18, 35] and also reacts with O_2_^−^ to generate •OH in the Haber-Weiss reaction [19, 35]. Glutathione peroxidase catalyzes H_2_O_2_ to form nonradical water and oxygen [35].

### 2.2. Xanthine Oxidoreductase

Xanthine oxidoreductase, the major source of superoxide in postischemic tissue too [4], consists of two interconvertible forms, xanthine dehydrogenase (XDH) preferably using NAD^+^ as an electron acceptor and xanthine oxidase (XO) using O_2_ as the terminal electron acceptor [30]. Xanthine oxidoreductase catalyzes the transformation of hypoxanthine and xanthine to uric acid, with O_2_^−^or H_2_O_2_ generation as by-products [41, 42]. Moreover, under acidic conditions (pH ~6.5), XDH may oxidize NADH instead of xanthine, thus promoting superoxide production RI, impaired autopha/RI [30]. Therefore, developing a sort of xanthine oxidoreductase inhibitors seems a promising intervention against MI/RI. But this approach sometimes gets nowhere due to differences in xanthine oxidoreductase abundance/activity between animal species [30]. For instance, application of XO inhibitor, like allopurinol, to the rabbit heart [43] and human heart [44] cannot protect against MI/RI, because the rabbit heart and human heart lack XO activity. However, activation of XO in hepatoenteric tissue of a rabbit, not in the heart, induced significant myocardial injury [45]. Perhaps because ROS released from extracardiac xanthine oxidase induce cardiac injury [35], and therefore, inhibition of extracardiac xanthine oxidase may be still an effective therapeutic treatment against oxidative stress-induced cardiac injury. In addition, xanthine dehydrogenase is converted to xanthine oxidase during ischemia [42]. In consideration that the capacity of superoxide production by XDH is regulated by the relative level of NAD^+^ to NADH, and the higher proportion of NADH, the more enhanced O_2_^−^ production [46], researches for finding an intervention to change the reductive state (low NAD^+^ to NADH ratio) during reperfusion period in the heart could be a feasible treatment to protect against MI/RI.

### 2.3. Uncoupled Nitric Oxide Synthase

Different effects of NO from various sources on MI/RI have been reported. NO derived from endothelial nitric oxide synthase and neuronal nitric oxide synthase are thought to protect against MI/RI, while inducible nitric oxide synthase-derived ones aggravate MI/RI and can also cause cardiac hypertrophy and oxidative stress [47]. NOS are enzymes containing flavin and heme and transfer electrons from the NADPH at the C-terminal (reductase domain) to the N-terminal heme (oxidase domain), reducing O_2_ and incorporating it into l-arginine to produce l-citrulline and NO [48, 49]. However, tetrahydrobiopterin (BH4), an essential cofactor of NOS, will be oxidized by ROS [50]. In the absence of l-arginine, BH4, or both, NOS can become a source of O_2_^−^ instead of NO [48, 51], thus becoming “uncoupled” to their primary role of NO synthesis and limiting the effect of NO in the vascular system. However, H_2_O_2_ could potentially induce and activate the eNOS [52, 53] that compensates for the reduction of NO due to the pathological processes above. Besides, it has been speculated that iNOS is increased under reperfusion stimulation and then synthesizes NO [54, 55]. NO exerts cytotoxic effects both in a direct way and via reacting with superoxide to form highly oxidizing agent peroxynitrite (ONOO^−^) that causes further cell and tissue damage [54, 56, 57].

### 2.4. Nicotinamide Adenine Dinucleotide Phosphate (NADPH) Oxidase

NADPH oxidases (NOXs) are a family of seven transmembrane electron transporters, respectively, named NOX1 to NOX5 and dual oxidase- (DUOX-) 1 and DUOX-2 [58, 59] that catalyze the transfer of electrons across biological membranes from the electron donor NADPH to O_2_, leading to the generation of O_2_^−.^[59] and, according to some reports, H_2_O_2_ [60–62]. To be more specific, it is believed that the dual oxidases and NOX4 predominately produce H_2_O_2_, while the remaining NOX isoenzymes largely produce superoxide [18, 27, 60]. The DUOX proteins are highly expressed mainly in the thyroid [59]. NOX3 is expressed almost exclusively in the inner ear [58]. The other NOX isoforms NOX1, NOX2, NOX4, and NOX5 are expressed in the cardiovascular system and are activated or highly expressed during myocardial ischemia or reperfusion [18, 63]. NOX2 and NOX4 are the main NOX subtypes that produce ROS especially O_2_^−^ and H_2_O_2_ in the heart, which promote oxidative stress RI, impaired autopha/RI [64]. NADPH oxidases are involved in other process of ROS production, such as the ROS generation by NOS and xanthine oxidase; NADPH oxidase-derived ROS may oxidize and degrade BH4 and activate xanthine oxidase [65, 66].

## 3. Pathways through Which Oxidative Stress Causes Myocardial Reperfusion Injury

**Figure 2:**
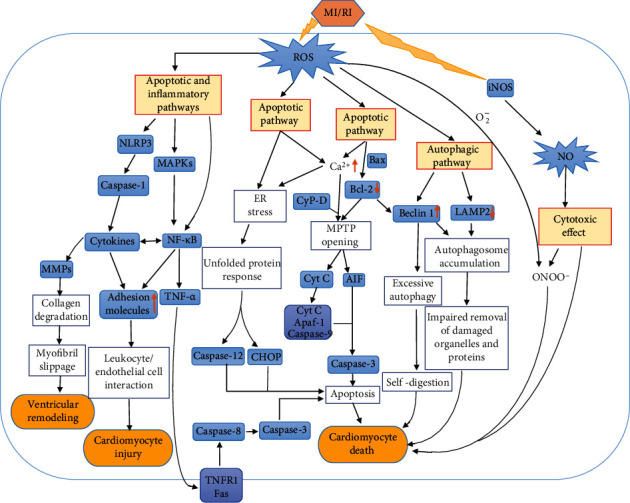
The damage of oxidative stress to cardiomyocytes during reperfusion. Reactive oxygen species affect Ca^2+^ overload and Bcl-2 family proteins, which lead to the mitochondrial permeability transformation pore opening and ultimately lead to myocardial apoptosis. Reactive oxygen species also trigger exogenous apoptosis by activating the MAPK family. Finally, reactive oxygen species initiate apoptosis through ER stress. Beclin1 and LAMP2, which are regulated by reactive oxygen species, cause impaired autophagy or excessive autophagy, thereby damaging cardiomyocytes. Via inflammatory response, reactive oxygen species induce pathological damage of the heart. NO, one of the members of reactive nitrogen, damages cardiomyocytes through direct cytotoxicity or generates ONOO^−^ with O_2_^−^ to cause cardiomyocyte damage. Abbreviations: MAPK: mitogen-activated protein kinase; NF-*κ*B: nuclear transcription factor-*κ*B; TNF-*α*: tumor necrosis factor-*α*; TNFR1: tumor necrosis factor receptor 1; Fas: tumor necrosis factor superfamily; CyP-D: cyclophilin D; Bcl-2: B cell lymphoma-2; MPTP: mitochondrial permeability transition pore; AIF: apoptosis-inducing factor; Cyt: cytochrome; LAMP2: lysosomal-associated membrane protein 2; Apaf-1: apoptosis protease-activating factor-1; ONOO^−^: peroxynitrite; NLRP3: nucleotide-binding oligomerization domain-like receptor protein 3; MMPs: matrix metalloproteinases; ER stress: endoplasmic reticulum stress; CHOP: CCAAT/enhancer-binding protein homologous protein.

Oxidative stress causes cell death either through directly destroying proteins, DNA, lipids, and other macromolecules or acting as a signal molecule in the cell death signaling pathway [67]. In this part, we mainly discussed the negative effects of ROS in reperfusion through apoptosis, autophagy, inflammation, and some other pathological process, along with the mechanisms involved (Figure 2).

### 3.1. Apoptosis of Myocardial Cells Induced by ROS

Apoptosis, a unique form of gene-regulated cell death, has been shown to be triggered or accelerated primarily during reperfusion or reoxygenation [68, 69]. Secondary necrosis (i.e., late apoptosis) of apoptotic cells can be caused by loss of membrane integrity of these cells [70], ROS-induced inactivation of caspases [71], and oxidant-induced failure of mitochondrial energy production [72]. It was thought that apoptosis might cause myocardial stunning [73], extension of infarction [74], cardiac dysfunction, and even heart failure [75, 76]. Mechanisms of apoptosis induced by ROS have been systematically described [34, 77].

Apoptosis triggered by disruption of mitochondrial homeostasis. Ca^2+^ is induced by ROS to influx into the cytoplasm and then influx into the mitochondria, resulting in the opening of MPTP, the collapse of mitochondrial membrane potential, and release of apoptotic signaling molecules such as cytochrome c and apoptosis-inducing factor (AIF) from the intermembrane space [34, 78–80]. In the cytosol, the apoptosome, formed with cytochrome c, apoptosis protease-activating factor-1 (Apaf-1), and caspase-9, activates caspase-3 ultimately initiating apoptosis [34, 79]. Evidence suggests that Bcl-2 prevents MPTP opening and inhibits caspase activity, thereby inhibiting apoptosis. On the contrary, Bax increased mitochondrial outer membrane permeability and caused the release of apoptotic factors [81, 82]. Beyond that mentioned above, oxidative stress also contributes to the translocation of the apoptotic protein Bax and Bad into the mitochondria where these factors form heterodimers with Bcl-2 [83], decreasing Bcl-2. A decreased Bcl-2/Bax ratio results in MPTP opening as well [79].

Apoptosis triggered by MAPK family. ROS activate MAPKs (primary p38 and JNK MAPKs), which mediate the dissociation of the NF-*κ*B from its inhibitor I*κ*B and upregulate activated NF-*κ*B [34]. NF-*κ*B in the cytoplasm influx into the nucleus, contributing to synthesis of TNF-*α* that releases to extracellular matrix, combines with membrane surface receptors (TNFR1 and Fas), activates caspase-8 and caspase-3, and then triggers an extrinsic death cascade [34]. ROS could also directly activate NF-*κ*B. H_2_O_2_ may directly mediate the dissociation of I*κ*B from NF-*κ*B and upregulate NF-*κ*B [84]. H/R-induced reactive oxygen intermediates activate NF-*κ*B via tyrosine phosphorylation of I*κ*B*α* [85]. Evidences of NF-*κ*B activation by oxidative stress have also been described by Bowie and O'Neill [86]. However, the activation of NF-*κ*B by H_2_O_2_ is cell-specific [86], and it has not been clarified whether the mechanism of activation above holds in cardiomyocytes.

Apoptosis induced by endoplasmic reticulum stress. RI, impaired autopha/RI, deficiency of glucose and nutrient supply, ATP depletion, ROS accumulation, and destruction of Ca^2+^ homoeostasis interfere with endoplasmic reticulum (ER) function, causing unfolded protein response with unfolded/misfolded protein accumulation, the condition referred to as ER stress [87, 88]. Numerous studies have indicated the association between ER stress and cardiomyocyte apoptosis [89]. Prolonged and/or excessive ER stress has been reported to induce ER-related apoptosis with increased expression of CCAAT/enhancer-binding protein homologous protein (CHOP) and the activation of caspase-12 [87, 90]. Simultaneously, ER stress was thought to disrupt the redox balance, causing ROS accumulation and mitochondria dysfunction, finally aggravating cardiomyocyte apoptosis [90].

#### 3.1.1. Effects of ROS on Intracellular Ca^2+^ Overload

Intracellular Ca^2+^ overload may be caused via multiple pathways that involved oxygen free radicals. Sarcolemmal Ca^2+^-ATPase related to the extrusion of Ca^2+^ from cardiac cells [91] and sarcoplasmic reticular Ca^2+^, Mg^2+^-ATPase sequestering Ca^2+^ from the cytoplasm into the lumen of sarcoplasmic reticulum [19] are altered by oxygen free radicals. ROS-induced intracellular Ca^2+^ overload is also reported to be caused via the activation of Na^+^/H^+^ exchanger [92], inhibition of Na^+^-K^+^ ATPase [92, 93], and enhancement of Na^+^/Ca^2+^ exchange (Ca^2+^ influx, Na^+^ efflux) [94], which all promote influx of Ca^2+^ into the intracellular space. Besides, increased extracellular Ca^2+^ influx is caused by ROS through membrane lipid peroxidation and the voltage-sensitive Ca^2+^ channel opening [4, 95].

#### 3.1.2. ROS Promote MPTP Opening

The formation of mitochondrial permeability transition pore (MPTP), an inner membrane nonselective pore, can cause ATP depletion, enhanced ROS production, membrane ion pump failure, solute entry, and then mitochondrial swelling, rupture, and release of apoptotic signaling molecules such as cytochrome c from the intermembrane space; this eventually leads to cardiomyocytes apoptosis, causing irreversible damage to the heart [4, 18]. The formation and opening of the MPTP are the main cause of mitochondrial dysfunction and cardiomyocyte death [96]. Therefore, prevention of MPTP opening with pharmacological interventions or genetic modifications has been reported to limit infarct size and decrease myocardial apoptosis and necrosis [97, 98]. MPTP have been reported [18] to be promoted by matrix Ca^2+^ and ROS but inhibited by low pH in ischemia. However, during reperfusion, restoration of pH, along with mitochondrial calcium overload and excessive ROS generation, causes the pore to form [18, 99], leading to cardiomyocyte death. In view of these influence factors, it is extremely significant to seek out some cardioprotective strategies that attenuate matrix calcium overload and oxidative stress or maintain a low pH during early phase of reperfusion [100–102]. In addition to stimulus above, matrix cyclophilin D (CyP-D) promotes MPTP opening via enhancing its calcium sensitivity [102], and binding of cyclophilin to the inner mitochondrial membrane could be greatly increased by oxidative stress [103]. CyP-D can be targeted by cyclosporin A (CsA) to protect cardiomyocytes [18]. However, the toxicity of cyclosporin [104] and even the adverse effects of Ca^2+^ efflux disorder in mitochondria after CyP-D-mediated MPTP opening is inhibited [105, 106] limit the functions of CsA and other CyP-D inhibitors. However, MPTP opening lasts only for the first few minutes after reperfusion [107] and may have little effect on Ca^2+^accumulation in the mitochondrial matrix. As for cyclosporine toxicity, more specific and novel CyP-D inhibitors may be explored.

#### 3.1.3. Activation of MAPK Family

Mitogen-activated protein kinases (MAPKs) are a protein family including extracellular signal-regulated kinases (ERK1 and ERK2), c-Jun N-terminal kinases (JNK1 and JNK2), and p38 MAPK, among which JNK and p38 MAPK promote apoptotic cell death, while ERK1/ERK2 exerts a protective effect [108]. However, the role of JNK MAPKs in apoptosis seems to be contradictory. ROS-dependent activation of JNK/p38 MAPKs has been shown to promote apoptosis [109]. JNK promotes apoptosis through the Bax subfamily of Bcl-2-related proteins [110]. But for ROS-induced apoptosis, JNK activation seems to represent a scavenger pathway for cells, which tries to escape apoptosis [111]. The contradictory role of JNK in apoptosis may lie in the difference in experimental procedures and the metabolic stage of the cells in different experiments [111]. In cardiac myocytes, JNK, ERKs, and p38 MAPK can be activated with the induction of ROS [112–114]. Hori and Nishida thought that apoptosis signal-regulating kinase 1, as an upstream signaling molecule, is activated by ROS, and then activates p38 and JNK, which finally causes apoptosis and cell hypertrophy [95]. The p38 MAPK, primarily related to contribution of apoptosis [111, 115, 116], is a key signal transduction factor mediating myocardial apoptosis following MI/RI [117]. For lung injury, p38 MAPK phosphorylates mitogen-activated protein kinase-activated protein kinase 2 (MK2), contributes the activation of caspase-3, and then leads to apoptosis and cell death [118]. Further study, nevertheless, is needed to determine whether this apoptotic pathway holds in cardiomyocytes. Another report has shown that p38-activated MK2 in MI/RI is detrimental to cardiomyocytes [119]. Although an important role of MK2 in inflammatory response has also been suggested in this literature [119], its regulation of TNF biosynthesis may not be contradictory with the MAPKs/NF-*κ*B/TNF-*α* apoptotic signaling pathway [34] previously mentioned. In the H/R model, p38 MAPK also regulates the accumulation of mitochondrial ROS [6]. TUNEL assay showed that inhibition of p38 kinase activity during hypoxia/reoxygenation prevented H/R-induced apoptosis [120]. On the contrary, activation of ERKs may protect cardiac myocytes from apoptotic death induced by oxidative stress [113]. ERKs also reduce ROS production by inhibiting NOX4 [121]. The dynamic balance between activation of JNK and p38 and activation of ERK may partly determine whether a cell survives or apoptosis [122].

#### 3.1.4. Endoplasmic Reticulum Stress

The ER, under physiological conditions, regulates many biological processes, including protein folding, assembly, modification and secretion, Ca^2+^ homeostasis, and lipid synthesis [123, 124]. However, when exposed to pathologic conditions, like ROS exposure, Ca^2+^ overload, deficiency of glucose and nutrient supply, and ATP depletion, homeostasis is impaired with the accumulation of unfolded/misfolded proteins, as described above [87, 88, 123]. RI, impaired autopha/RI, the unfolded protein response and/or ER-initiated apoptosis is likely to be triggered by both the depletion of oxygen and energy substrates and the subsequent sudden increase in oxygen free radicals [125]. It has also been reported that oxidative stress triggered by tissue reperfusion causes ER Ca^2+^ depletion, then leading to protein misfolding [126]. MI/RI-activated unfolded protein response upregulates multiple ER stress proteins, including chaperones glucose-regulated protein 78, activating transcription factor 6, and transcription factor X-box binding protein-1; many of which act as protective roles, alleviating the ER stress [127]. However, prolonged and/or excessive ER stress-triggered unfolded protein response may cause apoptosis through CHOP and the caspase-12 pathways [87, 90, 127]. CHOP has been known as a key upstream molecule of apoptosis mediated by ER stress. It also downregulates the expression of antiapoptotic Bcl-2 [124].

### 3.2. Autophagy of Myocardial Cells Induced by ROS

Autophagy is a major pathway for eukaryotic cells to degrade and recycle organelles and macromolecules by which cytosolic long-lived proteins and damaged organelles can be removed [128]. Organelles and macromolecules are sequestrated by double-membrane structures called autophagosomes, delivered to lysosomes, degraded by lysosomal hydrolases, and then recycled [129, 130]. Different from ischemic autophagy triggered by activation of the AMPK pathway and inhibition of the mTOR pathway [131–133], autophagy during reperfusion is upregulated by Beclin1 dependence [132]. On the contrary, reperfusion is accompanied by inactivation of AMPK [132] and mTOR activation [132, 134], and activated mTOR inhibits autophagy [134–136]. Enhancement of oxidative stress is both necessary and sufficient for causing autophagy in cardiomyocytes RI, impaired autopha/RI [137]. Autophagy can also be induced by Ca^2+^ overload [138], release of endoplasmic reticulum calcium [130], mitochondrial permeability transition pore (MPTP) opening [139], and CyP-D [140]. As mentioned above, these changes can be caused or enhanced by oxidative stress. Besides, the autophagy is also regulated by components of the apoptosis, including mitochondrial-localized Bcl-2 family members [141]. The antiapoptotic proteins Bcl-2 and Bcl-X_L_ inhibit autophagy through binding and inhibiting Beclin1 [142], while death-inducing Bcl-2 family members, such as Bcl-2 19 kDa interacting protein 3 or Bax, induce autophagy [141].

Autophagy is characterized by the protection of cell function under normal conditions, whereas autophagy under pathophysiological conditions can either protect against cell damage or serve as another form of programmed cell death, known as PCD type II [143]. Finding out whether autophagy activated respectively during ischemia and reperfusion plays a positive or negative role is important. It is thought that autophagy during acute myocardial ischemia and chronic hibernation is cardioprotective while it is detrimental in myocardial reperfusion after a short period of ischemia [132, 133, 144, 145]. Autophagy may itself be a physiological process that protects the heart by removing damaged mitochondria and other organelles and inhibiting ATP depletion. For instance, autophagy is suspected to reduce apoptotic damage via removing damaged mitochondria, thereby limiting the diffusion of proapoptotic factors like apoptosis-inducing factor, second mitochondria-derived activator of caspases, and cytochrome c, and reducing ATP depletion [146–149]. It can also repair myocardial cells injured during H/R by lysosomal autophagy removal of nonfunctional lysosomes [150]. However, Beclin1-dependent autophagy induced by reperfusion/reoxygenation only promotes autophagosome formation, while autophagosome clearance is impaired, resulting in impaired autophagic flux [151]. Impaired autophagy such as incomplete autophagic removal of damaged mitochondria [152], abnormal lysosome structure and accumulation of autophagic vacuoles [153], loss of lysosomal integrity, and lysosomal proteases released into the cytosol [150] may play a negative role in heart disease. Lysosomal-associated membrane protein 2 (LAMP2) is a key protein for autophagosome-lysosome fusion, and its expression is decreased during H/R injury [151], while BECN1 interferes with autophagosome-lysosome fusion and impairs autophagosome clearance [151, 154]. RI, impaired autopha/RI, impaired autophagosome clearance, which is mediated in part by the ROS-induced decrease of LAMP2 and the upregulation of BECN1 [151, 154, 155], leads to the accumulation of autophagosomes and abnormal clearance of damaged cell components, and finally forms a vicious cycle of increased ROS production and enhanced mitochondrial permeability [154]. Slightly different from the viewpoint of impaired autophagy, it is believed that reperfusion can lead to excessive autophagy, which is a cytotoxic effect that leads to excessive degradation and self-digestion of cellular constituents [136], resulting in irreversible damage and even cardiomyocyte death [132, 156]. In addition, in L929 cells, autophagy is activated by caspase inhibitors, which can lead to catalase degradation, intracellular ROS accumulation, membrane peroxidation, membrane integrity failure, and finally cell death [157].

### 3.3. Inflammation Triggered by ROS

Oxidative stress and inflammatory response are mutually promoting pathological processes [4, 65, 95]. For example, neutrophils act as one of the sources of ROS, while ROS from endothelial cells and cardiomyocytes amplify inflammatory response and influence nearby neutrophils, inducing a chain reaction of ROS generation [95]. Through the ischemia and reperfusion models of multiple organs, Toll-like receptors have been demonstrated to play an important bridging role in the interaction between oxidative stress and inflammatory response [158]. It is thought that ROS upregulate IL-1*β* through the NLRP3 inflammasome activation and caspase-1 expression [121]. IL-6 works as a downstream target of IL-1*β* [159]. It was demonstrated that thioredoxin-interacting protein-mediated NLRP3 inflammasome activation in cardiac microvascular endothelial cells was a novel mechanism of MI/RI [160]. In addition, NF-*κ*B, activated by ROS, regulates the expression of inflammatory genes, like IL-1*β*, IL-6, and TNF-*α* [161, 162].

The severe inflammatory conditions during MI/RI have been thought to occur due to the increased cytokines, that is, IL-6, IL-1*β*, and TNF-*α* [163]. During MI/RI, rapidly increased TNF-*α* exerts a negative effect via inducing the expression of adhesion molecules and chemokines, promoting the adhesion and interaction of leukocytes with endothelial cells, and increasing leukocyte infiltration [162, 164]. Similarly, IL-6 and IL-1*β* aggravate myocardial injury through promoting the adhesion of endothelial cells and neutrophils [162]. Beyond mentioned above, inflammatory factors have been reported to promote platelet adhesion, vascular endothelial injury, collagen exposure, and platelet activation [162]. The inflammatory response triggered by ROS during MI/RI has been reviewed in detail [165]. ROS can directly damage cardiomyocytes or promote cardiomyocyte injury through cytokine release, NF-*κ*B activation, increase of endothelial cell adhesion molecules, and leukocyte/endothelial cell interaction [165]. Besides, matrix metalloproteinases, activated by ROS and inflammatory cytokines, degrade collagens, then leading to myofibril slippage and left ventricular dilatation [95].

### 3.4. Other Pathophysiology Caused by ROS

As for oxidative stress-induced MI/RI, there are still other pathological changes that need to be further explored in addition to the frequently studied mechanisms mentioned above. For instance, ROS is believed to be involved in necrotic cardiomyocyte death via MPTP opening that is deemed as a main cause of the necrotic cell death rather than just inducing apoptosis [95]. Another report has shown that in cardiomyocytes, ROS activate NF-*κ*B and thereby inhibit the Nrf2-ARE pathway to promote oxidative stress-induced necrosis [166]. In addition to working as signaling molecules in the cell death pathways, ROS also initiate cell death through directly damaging various macromolecules like proteins, DNA, and lipids [67]. As was demonstrated, ROS-mediated reactions with proteins can inactivate key enzymes and ion transporters. With the peroxidation of polyunsaturated fatty acid of cell membranes, the permeability and selectivity of cell membranes to specific ions as well as receptor function alter [165]. Therefore, the effects of ROS on DNA, proteins, and lipids may be just another manifestation of the signaling pathways involved in cardiomyocyte damage. In addition to the above cell injury caused by ROS, NO, along with its product ONOO^−^, exerts cytotoxic effects that cause cell and tissue damage [54, 56, 57].

## 4. Interventions Targeting Oxidative Stress and Related Pathways in Myocardial Reperfusion

As mentioned above, based on the multiple sources of ROS, the effects of oxidative stress on MI/RI and the mutual influence of various pathways, it is extremely important to explore multiple targets and systematic intervening measures including traditional Chinese medicine to overcome the limitations of single therapy.

### 4.1. Antioxidants

It was demonstrated that oxidative stress to cardiomyocyte may lead to detrimental cellular effects like necrosis, apoptosis, or autophagy [22] and elicit MI/RI like arrhythmia, stunning, and infarction [167]. Suppression of OFRs accumulation during MI/RI can alleviate myocardial stunning, irreversible injury, and reperfusion arrhythmias [19]. Many therapeutic strategies have been developed to attenuate MI/RI through counteracting ROS generation and accelerating their consumption.

#### 4.1.1. Inhibition of ROS Generation

Amounts of potential sources of ROS have been researched, such as xanthine oxidase [25], NADPH oxidase (NOX) [26, 27], mitochondria [28], and uncoupled nitric oxide synthase [29]. Different treatments targeting multiple sources of ROS could reduce oxidative stress-induced injury in reperfusion.

*(1) Mitochondria*. Paraoxonase 2 binds specifically to the complex III in inner mitochondrial membrane, and its deficiency alters mitochondrial function partly by reducing the activities of mitochondrial complexes I/III, thus resulting in the superoxide generation and exacerbating the development of atherosclerosis [168]. A research aiming at exploring the regulatory role of paraoxonase 2 in MI/RI showed that paraoxonase 2 in the myocardium can reduce mitochondrial dysfunction and oxidative stress in cardiomyocytes by activating the PI3K/Akt/GSK-3 RISK pathway [169]. Based on the report that rapid reactivation of complex I, which generates H_2_O_2_ causing oxidative damage and the cell death, is a core pathological mechanism of MI/RI [170], mitochondria-targeted S-nitrosothiol, a mitochondria-selective S-nitrosating agent, can reversibly S-nitrosify complex I to slow the reactivation of mitochondria during the first minutes of the reperfusion, thereby decreasing ROS production and oxidative damage [170]. In adult Sprague-Dawley rat MI/RI model, pigment epithelium-derived factor decreased myocardial infarct size during MI/RI, downregulated myocardial apoptosis, improved cardiac function, and increased cardiac functional reserve [171]. The H9c2 myocardial cell hypoxia/reoxygenation (H/R) model was established to further study the protective mechanism of pigment epithelium-derived factor. The results indicate that the protective effect of pigment epithelium-derived factor on MI/RI is realized by inhibiting the production of mitochondrial and cytoplasmic ROS [171]. Dapagliflozin administration during MI/RI protects the heart by reducing infarct size, improving left ventricular function, and reducing arrhythmias. The protective effect is achieved in part by reducing mitochondrial ROS production and mitochondrial dysfunction [172].

*(2) Xanthine Oxidoreductase*. Xanthine oxidase inhibitors, which include purines (allopurinol and oxypurinol) and nonpurines (febuxostat and topiramate), exert antioxidant effects by reducing purine-derived ROS production [173]. In particular, the protective effects of allopurinol administration in MI/RI have been reported, including reduced infarct size, improved ventricular function, and reduced arrhythmia incidence [25, 174–176]. In a clinical trial examining the cardioprotective effect of oral allopurinol on patients receiving primary percutaneous transluminal coronary angioplasty after acute myocardial infarction, the results showed that allopurinol pretreatment could effectively inhibit the production of oxygen-derived radicals in myocardial reperfusion and restore left ventricular function [177]. Clinical researches showed that allopurinol improved cardiac function and reduced hospital mortality in patients undergoing coronary artery bypass surgery via inhibiting XO-derived ROS generation [178, 179].

*(3) NOS*. As mentioned above, the decreased bioavailability of NO caused by eNOS uncoupling and the cytotoxicity of NO synthesized by iNOS are the cause of myocardial damage induced by NOS. The depletion of tetrahydrobiopterin (BH4), an important cofactor of NOS, in MI/RI resulted in increased eNOS uncoupling and ROS production, but preischemia administration of liposomal BH4 can reduce the dysfunction of eNOS secondary to BH4 depletion during reperfusion, thus protecting cardiac function [180]. To investigate the effect of quercetin on the expression of NOX2, eNOS, and iNOS genes and proteins in the rabbit heart after myocardial ischemia/reperfusion injury, a MI/RI model was established in New Zealand white rabbits by myocardial ischemia for 30 min and reperfusion for 12 h. The results indicate that quercetin can not only inhibit MI/RI-induced expression of NOX2 and iNOS but also eNOS [181]. The protection to the myocardium by inhibiting eNOS expression may be due to the reduction of iNOS induction and decreased iNOS-derived peroxynitrite during late reperfusion, which is in contradiction with the positive effect of eNOS during early reperfusion [182]. Insulin [183] and pterostilbene [108] can not only increase the phosphorylation of eNOS, promoting the production of physiological NO and the reduction of superoxide, but also inhibit the expression of iNOS. It was shown that insulin and pterostilbene protected against MI/RI by blocking ONOO^−^-triggered oxidative/nitrative stress and that they both improved cardiac functions, as well as reduced myocardial infarction, apoptosis, and creatine kinase/lactate dehydrogenase release. Studies have shown that the exogenous donor of NO, NO-aspirin, releases NO at a rate similar to the endogenous NO derived from L-arginine, which limits infarct size, improves myocardial contractile dysfunction, and reduces the mortality rate, during MI/RI [184]. In addition, due to the antiplatelet aggregation and vasodilation effects of NO [184], it may reduce microvascular obstruction following reperfusion. Similar therapeutic effects may be achieved through microbubble oscillations, which can increase blood perfusion through activating the eNOS pathway and releasing NO [185].

*(4) NAD(P)H Oxidase*. The NOX family of NADPH oxidase may produce superoxide and other ROS by transporting electrons through the biological membranes from NADPH to O_2_ during MI/RI, especially NOX2 and NOX4 [64]. Injection with H_2_O_2_-responsive antioxidant PVAX nanoparticles was used to evaluate the therapeutic effect of PVAX on MI/RI in mice. The results of dihydroethidium staining showed that PVAX effectively inhibited MI/RI-caused ROS production, and PVAX targeted the production site of ROS, reducing the expression levels of NOX2 and NOX4 that are the main NOX subtypes in the heart, thus protecting against MI/RI [23]. By suppressing the PKC-б/NOX2 /ROS signaling pathways in H9c2 cells, Wenxin Granule, a Chinese patent medicine commonly used in cardiovascular diseases, inhibits oxidative stress, mitochondrial dysfunction, and myocardial cell apoptosis induced by H/R [186]. In addition, as has been demonstrated, cardiotonic pill that is a compound Chinese medicine used in the treatment of ischemic angina pectoris can achieve antioxidative effect by inhibiting NADPH oxidase activation, thus reducing MI/RI-caused rat myocardial injury and microcirculatory disturbance [187].

#### 4.1.2. Endogenous Antioxidant Systems

The endogenous antioxidant systems can protect cells against the potential injury through regulating the balance of individual ROS and their reactants, maintaining “redox homeostasis” [35]. Major endogenous antioxidants in cardiomyocytes include superoxide dismutase (SOD), catalase (CAT), glutathione peroxidase (GSH-Px), glutathione, coenzyme Q10 (ubiquinone), and vitamins C and E [35]. Some interventions enhance the activity of endogenous antioxidant systems. Cheng et al. evaluated the cardiac protective effect of N-propyl caffeamide, a newly synthesized caffeic acid derivative, on a mouse MI/RI model. The results showed that N-propyl caffeamide effectively reduced infarct size and the release of myocardial enzymes such as creatine kinase, creatine kinase isoenzyme, and lactate dehydrogenase. Biochemical analysis showed that N-propyl caffeamide increased the activity of antioxidant enzymes (such as CAT and SOD) while decreased the lipid peroxidation [188]. In a research to investigate the effects of galectin-3 on MI/RI, C57B6/J wild-type (WT) mice and galectin-3 knockout (KO) mice were used to establish murine model with MI/RI for 30 min of ischemia and 24 h of reperfusion. The results showed that troponin I in the galectin-3-KO group was significantly higher than that in the wild-type group, along with reduced SOD, GSH, and CAT and enhanced apoptotic activity [189]. Besides, trans sodium crocetinate was shown to upregulate sirtuin 3 expression and subsequently regulate the posttranslational protein modification of FOXO3a, thereby increasing SOD2 protein level and alleviating MI/RI-induced myocardial oxidative stress [190]. Silent information regulator 1 (SIRT1) can deacetylate and activate FOXO that synthesizes antioxidants such as manganese superoxide dismutase (MnSOD) and catalase [191, 192]. Berberine may target SIRT1 to protect against MI/RI induced by oxidative stress [193]. An in vitro MI/RI study showed that scutellarin, a flavone extracted from traditional Chinese medicine, could inhibit oxidative stress by increasing SOD concentration, thus protecting cardiomyocytes [194]. Intravenous administration of water-soluble acacetin prodrug could improve ventricular arrhythmias, infarct size, and cardiac dysfunction, which were induced by MI/RI in rats. Molecular mechanisms suggested that its protective effect on the myocardium was achieved partly by preventing the reduction of endogenous antioxidants such as SOD2 and thioredoxin [195]. A clinical research of 34 patients showed that human recombinant SOD alleviated reperfusion arrhythmias, but did not significantly improve left ventricular function. This may be due to the fact that the myocardial cell damage during reperfusion is not caused by superoxide but by other ROS, or that SOD in the damaged myocardium does not reach the effective concentration during reperfusion [196]. Another clinical report has shown that N-acetylcysteine, a precursor of glutathione, limits infarct size, reduces reperfusion ventricular arrhythmias, improves global and regional left ventricular function, and normalizes electrocardiogram [197]. In addition, there is evidence that preoperative upregulation of antioxidant enzymes and nonenzymatic antioxidants may reduce the incidence of postoperative atrial fibrillation [22].

#### 4.1.3. Exogenous Antioxidants

Since endogenous antioxidant levels are not sufficient to prevent reperfusion injury [198], it is also important to explore more exogenous antioxidants. Pretreatment for the hearts with N-acetylcysteine and N-mercaptopropionylglycine that are exogenous antioxidants has been shown to be beneficial in preventing MI/RI, but further studies are needed to determine their effectiveness in reversing MI/RI-induced abnormalities in the heart [199]. Tong et al. used in vitro H/R cell model and in vivo local MI/RI mouse models to explore the effects of intravenous administration of lycopene on ROS during MI/RI. It suggested that intravenous administration of lycopene could protect mice from MI/RI by inhibition of ROS accumulation [200]. It was reported that novel pyridoindole derivatives seemed to inhibit the incidence of reperfusion injury like ventricular tachycardia and ventricular fibrillation in MI/RI through antioxidation and free radical scavenging protection. In addition, SMe1EC2, one of the pyridoindole derivatives, promotes recovery of the left ventricular function, such as decreasing left ventricular end-diastolic pressure and recovery of the stunned myocardium [201]. Besides, probucol, a lipophilic antioxidant, has been reported to reduce myocardial stunning during reperfusion following short-term ischemia in the rabbit [202].

#### 4.1.4. Mitochondria-Targeted ROS Scavengers

Antioxidants that are neither targeted nor accumulated in the mitochondria may be ineffective [10]. Mitochondria are deemed the major intracardiac source of ROS during MI/RI [35]. Mitochondria-targeted ROS scavengers may be more effective and more easily controlled than general antioxidants. MitoQ [203, 204], a mitochondria-targeted antioxidant containing the antioxidant quinone moiety, can be concentrated by the lipophilic triphenylphosphonium cation for several hundredfold within mitochondria, protecting the cardiomyocytes from MI/RI. Mitochondria-targeted Szeto-Schiller peptides (SS peptides) can decrease mitochondrial ROS generation, prevent mitochondrial permeability transition, inhibit cytochrome c release, reduce lipid peroxidation, limit the infarct size, prevent reperfusion ventricular arrhythmias, restore myocardial contraction, and prevent reperfusion-related myocardial stunning [205, 206]. Treatment of irisin, a muscle-origin protein, reduces infarct size, improves left ventricular ejection fraction, decreases serum troponin I, and inhibits apoptosis during MI/RI by regulating mitochondrial localization of SOD2 and increasing SOD2 activity [207]. Furthermore, since rapid reoxidation of succinate by succinic dehydrogenase may lead to massive ROS generation and cardiomyocyte death during reperfusion [208], inhibition of succinic dehydrogenase by malonate limits infarct size via alleviating ROS generation in the isolated mouse hearts during reperfusion period [208].

#### 4.1.5. Regulation of the Oxidation Defense System

*(1) Nuclear Factor Erythroid-Related Factor 2 (Nrf2)*. Nrf2, a nuclear transcription factor, plays an indispensable regulatory role in the defensive genes that encode detoxifying enzymes and antioxidant proteins, contributing to cellular resistance to oxidants [209, 210] and it is involved in inducing endogenous antioxidant enzymes to respond to oxidative stress [211]. Nrf2 binds to its cytosolic repressor Kelch-like ECH-associated protein 1 under nonoxidative stress and is ultimately targeted for ubiquitination and proteasomal degradation. However, Nrf2 dissociates from Kelch-like ECH-associated protein 1 and migrates to the nucleus under the influence of ROS. After binding to antioxidant response elements (AREs), Nrf2 promotes the expression of antioxidant genes and produces enzymes related to antioxidant defense, such as glutathione reductase (GR), heme oxygenase 1 (HO-1), and superoxide dismutase 1 (SOD1) [18, 209, 210, 212]. HO-1, an intracellular inducible phase II detoxifying enzyme, can be regulated by Nrf2 [213]. The Nrf2/HO-1 signaling pathway is related to defense against a variety of oxidative-inducing agents and represents a promising target for inhibiting MI/RI [213]. Many drugs or extracts thereof, such as resveratrol [211] and triptolide [214], inhibit oxidative stress by activating the Nrf2/HO-1 signal, thereby limiting infarct size and improving cardiac function. Preconditioning with Potentilla reptans L. root can exert cardioprotective and antiapoptotic effects through NO release, the Nrf2 pathway, and endogenous antioxidant activity, thereby alleviating arrhythmias and infarct size, and improving myocardial stunning [215]. Upstream signaling molecules of Nrf2 can also serve as targets for MI/RI therapies, such as phosphatidylinositol-3-kinase (PI3K) [216, 217], protein kinase C (PKC) [217], and silent information regulator 1(SIRT1) [218]. Glycogen synthase kinase 3 (GSK3) [219–221], which promotes degradation and inactivation of Nrf2, is also a therapeutic target. For instance, Shanmugam et al. used the Langendorff isolated heart perfusion system to research the myocardial protective effect of fisetin, a natural flavonoid, on MI/RI. The results suggested that fisetin could induce the expression of Nrf-1/2 through inhibiting GSK-3, thereby synthesizing HO-1, SOD, and GR, clearing ROS, and finally inhibiting apoptosis [222].

*(2) Hypoxia-Inducible Factor-1 (HIF-1)*. HIF-1 is a critical regulator of the transcriptional response to hypoxia conditions of mammalian cells [223]. Evidences suggested that HIF-1 expression is increased after myocardial ischemia [224, 225] and that HIF-1 is also activated by ROS [226, 227]. HIF-1 protects the myocardium from reperfusion injury. For example, HIF-1*α* mediates ischemic preconditioning [18, 228], regulating HO-1, and eNOS expressing [18], which, as described previously, alleviate MI/RI-induced oxidative stress. HIF-1 is regulated by PI3K/Akt. For instance, troxerutin alleviates oxidative stress in H9c2 cardiomyocytes through activation of the PI3K/Akt/HIF-1*α* signaling pathway [229]. Besides, the antioxidants N-acetylcysteine and allopurinol combat MI/RI synergistically in diabetes primarily restore through the HIF-1*α*/HO-1 signaling pathway [230].

*(3) PI3K/Akt*. Activation of phosphatidylinositol-3-kinase (PI3Ks) and its downstream target protein kinase B (Akt) regulates myocardial oxidative stress and promotes myocardial protection against MI/RI [231]. The regulatory effect of insulin on NOS mentioned above may be realized through the PI3K/Akt-dependent pathway [183]. GSK-3, which promotes MPTP [134], inhibits activation of mTOR [134], and promotes Nrf2 degradation [219–221], is inactivated by Akt phosphorylation [232]. Pretreatment with hydroxytyrosol seems to reduce myocardial infarct size, decrease apoptosis, and improve cardiac systolic function through this signaling pathway during MI/RI [233]. In addition, Akt acts on cytoplasmic peptide Bad (Bcl-2 family proteins), which is isolated in the cytoplasm, and inhibits apoptosis [232]. Signaling molecules that regulate the PI3K/Akt pathway can be used as targets for antioxidative stress. For example, phosphatase PTEN that negatively regulates the PI3K/Akt pathway can be inhibited by ROS [234, 235], exosomes derived from bone marrow stromal cells [236], or Achyranthes bidentata polypeptides [237], then enhancing Akt phosphorylation and promoting myocardial protection. Treatments targeting PI3K/Akt or related signaling pathways reduce reperfusion complications such as arrhythmias [238, 239], myocardial stunning [239], myocardial no-reflow [240], and adverse remodeling [241].

### 4.2. Regulation of Apoptosis-Related Pathways

#### 4.2.1. Regulation of Ca^2+^ Overloading

Intracellular Ca^2+^ overload, as mentioned above, can be triggered by ROS in a variety of ways [19, 91–94]. Apart from inducing MPTP opening, Ca^2+^ overload in cardiomyocytes also leads to cell death by causing excessive contraction of cardiomyocytes during reperfusion [5]. Therefore, the exploration of strategies to inhibit Ca^2+^ overload may provide new ideas for the prevention and treatment of oxidative stress-induced reperfusion injury. For example, intracardiac injection of 4(RS)-4-F4t-neuroprostane before ischemia, a nonenzymatic oxidized metabolite of cardiac protective docosahexaenoic acid, can limit myocardial infarction and reduce the incidence of ventricular arrhythmias [242]. The results not only showed the protective effect of 4(RS)-4-F4t-neuroprostane against mitochondrial Ca^2+^ overload, which is related to decreased MPTP opening, but also suggest that its antiarrhythmic properties seem related to reduced Ca^2+^ release from sarcoplasmic reticulum, diastolic membrane hyperpolarization, and action potentials shortening [242]. Tetramethylpyrazine, an alkaloid extracted from the traditional Chinese medicine Ligusticum chuanxiong Hort, protects against MI/RI by preventing Ca^2+^ overload and scavenging OFRs, among other beneficial pathways [243]. Mitochondrial ATP-sensitive potassium channel (mitoK_ATP_) can reduce mitochondrial Ca^2+^ overload, increase ATP synthesis, and increase protective ROS production during preconditioning but decrease ROS generation during reperfusion [244, 245]. Activation of mitochondrial potassium channels partly protects against reperfusion injury by a mild depolarization and reduction of Ca^2+^ accumulation in the matrix, then reducing MPTP opening and apoptosis [246]. The novel H_2_S donor 4-carboxyphenyl isothiocyanate can prevent Ca^2+^ accumulation in the mitochondrial matrix, and the mitoK_ATP_ may be a relevant pharmacological target of it [247]. Regulation of Ca^2+^ has been reported to ameliorate complications associated with MI/RI. In a model simulating MI/RI, Salvia miltiorrhiza was showed to improve antioxidant and calcium regulation in cardiomyocytes during MI/RI and reduce arrhythmias and apoptosis [248]. In a porcine model of MI/RI, sarcoplasmic reticulum Ca^2+^ ATPase pump improves mechanical and electric stability in the heart through reducing Ca^2+^ overload and then inhibits ventricular arrhythmias [249]. In addition, calcium regulation can reduce myocardial death and heart failure [250].

#### 4.2.2. Regulation of MPTP Opening

Mitochondria-mediated apoptosis is recognized as a key part in MI/RI [251]. During reperfusion, Ca^2+^ overload and excessive ROS production, along with some other factors, can trigger MPTP opening [252]. Myocardial ischemia/reperfusion injury mainly depends on the opening of MPTP within the first few minutes of reperfusion and subsequent mitochondrial dysfunction [253, 254]. MPTP contributes 50% to MI size [253]. It has been reported [254] that ischemic preconditioning and ischemic postconditioning may inhibit MPTP formation by regulating calcium balance, oxidative stress, ATP level, and pH recovery, as well as direct MPTP inhibition involving the complex signal transduction pathways, thus exploring more targets for the treatments of myocardial reperfusion injury. In the rat MI/RI models established by ligation of the left coronary artery (30 min) and reperfusion (120 min), pretreatment of 3-methyl-1-phenyl-2-pyrazolin-5-one, a free radical scavenger, can inhibit intracellular Ca^2+^ overload caused by oxidative stress, thereby inhibiting MPTP opening [198]. In addition, mitochondrial swelling, the release of cytochrome c, and myocardial cell apoptosis can be reduced by 3-methyl-1-phenyl-2-pyrazolin-5-one through inhibiting MPTP opening [198]. Schaffer et al. have comprehensively reviewed the protective effects of taurine in MI/RI, which suppresses MPTP activation by inhibiting calcium overload, reducing ROS overproduction in respiratory chain, and activating the Akt-dependent protective signaling pathway [255]. It has been reported with heart protection during bypass surgery, heart transplantation, and myocardial infarction, and severe loss of it may increase the risk of reperfusion ventricular remodeling and heart failure [255]. Inhibition of MPTP during reperfusion was also showed to promote functional recovery and reduce mortality in mice [256]. Besides, matrix CyP-D promotes MPTP opening by enhancing its calcium sensitivity [102], and CyP-D serves as a target for cyclosporin A to protect cardiomyocytes [18]. However, Bennett and Norman described toxicity of cyclosporine [104]. Newer CyP-D inhibitors such as hematopoietic-substrate-1-associated protein X-1 [98] and polylactic/glycolic acid nanoparticles [257] may overcome the shortcoming of CsA. Cardio-specific hematopoietic-substrate-1-associated protein X-1 downregulates cyclophilin D levels through interfering its binding to heat shock protein-90 [98], rather than involving CsA. Polylactic/glycolic acid nanoparticles regulate the in vivo kinetics of CsA by selective delivery to the MI/RI-related cardiomyocyte cytosol and mitochondria [257], and treatment with nanoparticles incorporated with CsA enhanced the cardioprotection of CsA, even at low CsA concentrations [257], which may effectively reduce the toxicity of CsA. In addition, increased acetylation of CyP-D after myocardial reperfusion promotes MPTP opening, and ischemic postconditioning may promote deacetylation of CyP-D and prevent MPTP opening by increasing sirtuin 3 activity which prevent lethal reperfusion injury [258].

#### 4.2.3. Regulation of Bcl-2 Family Proteins

ROS triggers the initiation of apoptosis, which is related to the decrease of antiapoptotic Bcl-2 and proapoptotic Bax ratio during MI/RI [259]. The ratio of Bcl-2 to Bax can decide whether cell survives or not following apoptotic stimulation [260]. Therefore, the exploration of therapeutic strategies regulating the expression of Bcl-2 and Bax may provide new targets against oxidative stress. For example, the cardiac protective effect of N-propyl caffeamide is not only realized by regulating the activity of CAT and SOD as mentioned above but also by upregulating the expression of Bcl-2 in myocardial tissue and inhibiting the expression of Bax [188]. In addition, it has been reported that treatment with diltiazem plus SOD during MI/RI in a rat model can inhibit the apoptosis pathway, and that one of the results showed increased Bcl-2 expression and decreased Bax expression [261]. The phenylethanoid glycoside-rich extract of Cistanche deserticola reduces oxidative stress in the reperfusion myocardium and plays an important role in suppressing apoptosis pathways. Results of the research also included upregulation in the ratio of Bcl-2 to Bax [262]. It has been shown that adrenomedullin can protect against MI/RI-induced myocardial infarction, arrhythmias, and apoptosis by activating NO/cGMP to inhibit ROS-induced phosphorylation of Bax and p38 MAPK and activation of the Akt-Bad-Bcl-2 signaling pathway [263].

#### 4.2.4. Regulation of MAPK Family

Activated by ROS, MAPKs (mainly p38 and JNK MAPKs) initiate exogenous cell death cascades through the MAPK/NF-*κ*B/TNF-*α* signaling pathway. Interventions targeting p38 MAPK or JNK have been shown to protect against oxidative stress-induced MI/RI. Dual-specificity phosphatase is upregulated by N-acetylcysteine pretreatment [120]. And the modulation of p38 MAPK by dual-specificity phosphatase is indispensable to improve cardiovascular function under oxidative stress [120]. Betulinic acid protects H9c2 cells from MI/RI by inhibiting oxidative stress and apoptosis, and the Nrf2/HO-1, JNK, and p38 pathways are involved in mediating these protective effects [264]. Syringic acid, a natural O-methylated trihydroxybenzoic acid monomer that is extracted from Dendrobium nobile Lindl., protects H9c2 cardiomyocytes from H/R-induced apoptotic injury via inhibiting the activation of the p38 MAPK and JNK signaling pathways [265]. Injection of snakegourd peel, a traditional Chinese herbal medicine, inhibits the apoptosis of myocardial cells by reducing intracellular Ca^2+^ overload, inhibiting the activation of caspase-3, and downregulating the phosphorylated JNK (p-JNK) and p38 MAPK (p-p38 MAPK) protein expression [266].

#### 4.2.5. Regulation of Endoplasmic Reticulum Stress

During MI/RI, prolonged or excessive ER stress triggered by oxidative stress and other causes acts as a target for intervening in oxidative stress-induced reperfusion injury; in that, it causes apoptosis of the cardiomyocytes. Transforming growth factor *β*-activated protein kinase 1, a key regulator of cell death, may trigger MI/RI as an upstream signaling molecule of the ROS/ER stress pathway, and inhibition of it significantly decreased MI/RI-induced infarct size, reduced cell death, and improved cardiac function [87]. It has been reported that prevention of the ER stress with two chemical chaperones, tauroursodeoxycholic acid and 4-phenylbutyric acid, could limit the deterioration of the contractile function in the stunned myocardium such as reperfusion following acute myocardial infarction [88]. In vitro and in vivo experiments have shown that sulodexide pretreatment [267] and tournefolic acid B [268] may exert inhibiting role in ER stress through the PI3K/Akt pathway and then inhibit cell apoptosis. Silibinin treatment has been reported to improve cardiac function, reduce infarct size, and inhibit fibrotic remodeling in MI/RI mice. Its protective effect was achieved partly by inhibiting ER stress [90]. Shuxuening injection, an extract of Ginkgo biloba, protects against MI/RI mainly by preventing oxidative stress and ER stress, thus regulating the Toll-like receptor 4/NF-*κ*B pathway so as to reduce inflammation, and inhibiting procoagulant-related factors to reduce thrombosis [162].

### 4.3. Regulation of Autophagy-Related Pathways

The decline of LAMP2 and upregulation of BECN1 mediate the damage of autophagosome clearance during MI/RI, forming a vicious cycle of increased ROS production and enhanced mitochondrial permeability, which eventually leads to cell death [151, 154, 155]. Therapeutic measures targeting BECN1 or LAMP2 expression may reduce oxidative stress-induced reperfusion injury, such as partial BECN1 knockdown [154], inhibition of BECN1 expression by urocortin [269], and upregulating LAMP2 by exogenous calreticulin postconditioning [270]. There are other therapies that do not rely on Beclin1 and LAMP2 to restore impaired autophagy. For example, sustain-releasing H_2_S donor 5-(4-methoxyphenyl)-3H-1, 2-dithiole-3-thione [155], metformin [271], and sevoflurane precondition [272] restore the impaired autophagic flux induced by MI/RI through AMPK activation, thus protecting the myocardium from MI/RI. Intermittent fasting [273] and cilostazol [274] protect against myocardial ischemia/reperfusion injury by transcription factor EB-mediated transcriptional initiation of autophagy-lysosome mechanisms. Transient hypoxia can also protect against reperfusion injury by slightly upregulating autophagy [275]. In a MI/RI model with female farm pigs, myocardial hypothermia was shown to prevent myocardial remodeling through increasing autophagic flux and mitophagy [276]. Since excessive autophagy caused by MI/RI leads to cytotoxic effects with excessive degradation of cellular components and self-digestion, prevention of excessive autophagy caused by MI/RI may play a protective role in the heart. For example, PI3K/Akt upregulates mTOR expression, promotes downregulation of autophagy, and protects myocardial reperfusion injury [136]. Trimetazidine inhibits excessive autophagy induced by MI/RI by activating the Akt/mTOR pathway, which protects the rat hearts from heart failure, reduces infarct size, and so on [136]. Sevoflurane postcondition protects the rat heart from MI/RI by inhibiting autophagy overactivation and promoting autophagosome clearance [277]. Phellinus linteus mycelium pretreatment was used to significantly reduce MI/RI-induced myocardial infarct size, lactate dehydrogenase level, ventricular arrhythmias, and mortality in part by enhancing protective autophagy and inhibiting excessive autophagy [278].

### 4.4. Regulation of Inflammatory Response

The nuclear transcription factor-*κ*B may be an ideal target for the treatments against reperfusion injury; in that, it plays an important role in cardiomyocyte apoptosis and inflammatory injury triggered by oxidative stress during MI/RI. Apart from inhibition of ER stress, silibinin also exerts cardioprotective effect against MI/RI through alleviating inflammatory response via deactivating the NF-*κ*B pathway [90]. Researches show that microRNA-130a-5p targeted high mobility group box 2 to downregulate the NF-*κ*B to relieve the MI/RI-induced inflammatory injury [279]. Cytokines such as TNF-*α*, IL-6, and IL-1*β* are also indispensable in ROS-induced inflammatory injury. Dietary selenium intake, for example, reduces myocardial infarct size of rats and reduces adverse remodeling, and beneficial effect of it might be partly related to the inhibition of proinflammatory cytokine overexpression [280]. The supplementation of phyllanthin, a major bioactive lignin compound from *Phyllanthus* species, has also been reported to diminish IL-6, IL-1*β*, and TNF-*α* in the mice during MI/RI, as well as suppress the overexpression of NF-*κ*B [163]. In addition, interventions acting on inhibiting NLRP3 inflammasome activation may be deemed as novel therapies for relieving MI/RI [160].

## 5. Conclusion

Reperfusion following myocardial ischemia may lead to accelerated myocardial injury and worsening clinical outcomes. One of the most important pathological mechanisms in reperfusion injury is oxidative stress, which is the imbalance between the antioxidant system and the excessive production of ROS, leading to the toxic accumulation of reactive oxygen intermediates.

Multiple sources of ROS have been reported, including electron leakage from the ETC in mitochondria, the conversion of hypoxanthine and xanthine to uric acid catalyzed by xanthine oxidoreductase, “being uncoupled” of NOS to their primary role of NO synthesis, the transfer of electrons from NADPH catalyzed by NADPH oxidase, and other sources like the monoamine oxidases, lipoxygenases, cyclooxygenases, cytochrome P450, neutrophils, and catecholamine.

ROS mainly cause cardiomyocyte death through apoptotic pathway, autophagic pathway, inflammatory pathway, and cytotoxic effect. On the one hand, ROS initiate one apoptotic pathway through Ca^2+^ overload and decreased ratio of Bcl-2 to Bax, then formation of MPTP, mitochondrial membrane potential collapse, release of apoptotic signaling molecules, and finally activation of caspase-3. On the other hand, ROS initiate exogenous apoptotic pathway by activating MAPK family, upregulating activated NF-*κ*B, promoting the synthesis and release of TNF-*α*, binding of TNF-*α* to membrane surface receptors, and activating caspase-8 and caspase-3. Besides, ROS trigger ER-related apoptosis with the increased expression of CHOP and the activation of caspase-12. As for autophagic pathway, ROS induce decrease of LAMP2 and upregulation of BECN1, leading to impaired autophagic flux and excessive autophagy. About inflammation, ROS lead to pathological injury through inflammatory response with cytokine release, NF-*κ*B activation, increased adhesion molecules, and leukocyte/endothelial cell interaction. The inflammatory response also induces MMPs activation, then leading to collagen degradation, myofibril slippage, and left ventricular dilatation. Finally, NO synthesized by iNOS and ONOO^−^ produced by NO and O_2_^−^ cause cardiomyocyte death through cytotoxicity.

Fortunately, some interventions are currently available for ROS-induced reperfusion injury, including antioxidant therapy, along with regulation of apoptosis-related pathways, autophagy-related pathways, and inflammatory response. Researches have shown that these interventions can reduce reperfusion-mediated complications such as remodeling, arrhythmias, myocardial stunning, microvascular obstruction, and heart failure to a certain extent, as well as reduce mortality.

However, researches on the mechanisms of reperfusion injury caused by oxidative stress still need to be further improved, and more measures targeting various pathways and targets need to be explored. In addition, many of the current studies are based on animal or molecular level while fewer clinical studies have been done. Therefore, in future studies, (i) it may be of additional value to develop a comprehensive understanding of the oxidative stress involved in MI/RI and its associated signaling pathways, thereby reducing the risk of exposure. (ii) In terms of treatments, in addition to antioxidant therapies, systematic interventions targeting various pathways may overcome the limitations of single measures and targets. (iii) In addition to more abundant and mature laboratory research, more clinical research should be performed appropriately.
